# Smoking and Smoking Cessation in the Risk for Fetal Growth Restriction and Low Birth Weight and Additive Effect of Maternal Obesity

**DOI:** 10.3390/jcm9113504

**Published:** 2020-10-29

**Authors:** Małgorzata Lewandowska, Barbara Więckowska, Lidia Sztorc, Stefan Sajdak

**Affiliations:** 1 Medical Faculty, Lazarski University, 02-662 Warsaw, Poland; 2 Division of Gynecological Surgery, University Hospital, 33 Polna Str., 60-535 Poznan, Poland; ssajdak@ump.edu.pl; 3 Department of Computer Science and Statistics, Poznan University of Medical Sciences, 60-806 Poznan, Poland; barbara.wieckowska@ump.edu.pl; 4 Akademisches Lernkrankenhaus der Charité, Oberhavel Kliniken, Klinik Oranienburg, 16515 Oranienburg, Germany; lidia.sztorc@gmx.de; 5 Caritas-Klinik Dominikus, Berlin Reinickendorf, 13467 Berlin, Germany

**Keywords:** smoking cessation, paradoxical effect, pregnancy, birth weight, fetal growth, obesity

## Abstract

Many studies have shown that neonates of smoking mothers have a lower birth weight, but several issues remain poorly studied, e.g., the effects of giving up smoking or the combined effects of smoking and maternal obesity. Therefore, we evaluated a prospective cohort of 912 mothers in a single pregnancy, recruited in Poland, in 2015−2016. In the cohort, we recorded 72 (7.9%) newborns with birth weight <10th percentile, 21 (2.3%) fetal growth restriction (FGR) cases, and 60 (6.6%) low birth weight (LBW, <2500 g) newborns. In the cohort, 168 (18.4%) women smoked before pregnancy; the mean number of cigarettes/day was 10.8 (1–30), and the mean number of years of cigarette smoking was 8.5 (1–25). Among smokers, 57 (6.3%) women smoked in the first trimester. Adjusted odds ratio (AOR) of newborn outcomes (with 95% confidence intervals, CI) was calculated in multi-dimensional logistic regressions. Compared to participants who had never smoked, smoking before pregnancy was associated with a higher odds ratio of birth weight <10th percentile (AOR = 1.93, CI: 1.08–3.44, *p* = 0.027), but the result for LBW (AOR = 2.76, CI: 1.05–7.26, *p* = 0.039) and FGR (AOR = 1.13, CI: 0.38–3.36, *p* = 0.822) had the wider confidence interval or was insignificant. Effects of smoking cessation before pregnancy were statistically insignificant for the studied outcomes. Smoking in the first trimester was associated with a higher risk of birth weight <10th percentile (AOR = 4.68, CI: 2.28–9.62, *p* < 0.001), LBW (AOR = 6.42, CI: 1.84–22.36, *p* = 0.004), and FGR (AOR = 3.60, CI: 0.96–13.49, *p* = 0.057). Smoking cessation in the second/third trimester was associated with a higher odds ratio of birth weight <10th percentile (AOR = 4.54, CI: 1.58–13.02, *p* = 0.005), FGR (AOR = 3.36, CI: 0.6–18.74, *p* = 0.167), and LBW (AOR = 2.14, CI: 0.62–7.36), *p* = 0.229), to a similar degree to smoking in the first trimester. The odds ratios were higher in the subgroup of pre-pregnancy body mass index ≥25 kg/m^2^ for the risk of birth weight <10th percentile (AOR = 6.39, CI: 2.01–20.34, *p* = 0.002) and FGR (AOR = 6.25, CI: 0.86–45.59, *p* = 0.071). The length of cigarette smoking time was also the risk factor for studied outcomes. Conclusions: Smoking in the first trimester increased the studied risks, and the coexistence of excessive maternal weight increased the effects. Smoking cessation during the second/third trimester did not have a protective effect.

## 1. Introduction

In recent decades, attention has been paid to factors impacting the fetal environment as important elements of the concept of the development of health and disease (DOHaD) [1,2]. Adverse birth outcomes have been associated not only with increased perinatal mortality and offspring neurodevelopmental disorders but also with long-term adverse health effects [1,3]. In particular, abnormal (low and high) birth weight has been associated with a higher risk of “non-communicable diseases” such as obesity/overweight or diabetes mellitus and cardiovascular diseases [1,2,3,4,5].

Cigarette smoking is a modifiable environmental factor associated with many adverse pregnancy results, such as low birth weight, preterm birth, and higher newborn mortality [2,4,6,7,8]. Tobacco smoke contains several thousand chemicals that can be harmful to the placenta and pass through the placenta to the fetus [2,3]. An important component of tobacco smoke, nicotine can interfere with the physiological transformation of spiral arteries and increase the risk of pregnancy complications [4,9]. On the other hand, as has been demonstrated, nicotine has an anti-inflammatory effect mediated by binding to the acetylcholine receptor (with the α7-nAChR subunit) and inhibiting the production of proinflammatory cytokines, which may be responsible for the protective effect of nicotine in processes stimulated by placental ischemia or inflammatory factors [10,11]. Toxic substances from tobacco smoke increase inflammation and oxidative stress, which can reduce levels of antioxidants.

Mechanisms of the relations between smoking during pregnancy and inappropriate weight at birth have not been explained yet. The effect of multi-direction impacts of different components of cigarette smoke on complex multi-factor processes associated with the fetal growth are difficult to assess [12,13]. In addition to genetic factors, the main factors affecting fetal growth include the placenta, which guarantees the flow of nutrients to the fetus [14,15,16], as well as the mother’s nutritional status [13] and environmental factors [14]. Blood flow reductions in placenta, placental ischemia, hypoxia, and intensified oxidative stress and inflammation have been identified as key elements of pathogenesis of adverse fetal outcomes. Lower levels of antioxidants in maternal serum have been associated with a higher risk of FGR [12] and birth weight <10th percentile [13].

Although many studies have shown that neonates of smoking mothers have a lower birth weight [5,17], not everything has been clarified. Firstly, the effects of smoking cessation on birth weight have not been established [18,19]. It is important to explore this subject because it was found that smoking cessation during pregnancy can be associated (paradoxically) with increased risk of other pregnancy complications (e.g., preeclampsia and isolated gestational hypertension) [20]. Secondly, studies on the combined effects of smoking and maternal obesity are few and the results divergent [21,22,23]. Mitigation of the effects of smoking in obese mothers was found earlier and was explained by the relation of obesity with excessive fetal growth (and macrosomia) [21,22]. However, according to other research, obesity increases the risk of fetus growth reduction (not only macrosomia) and the risk of other pregnancy complications associated with placenta flow impairment [24,25]. This could suggest that obesity can also intensify the relations of smoking to fetus growth reduction. Thirdly, an analysis of the available literature indicates that the studies often cover one category of newborn weight [14,15,26].

The purpose of our study was a detailed analysis of nine categories of smoking (reported by the mother), including smoking before or during pregnancy and smoking reduction or cessation during pregnancy. We assessed smoking as categories and the amount of smoking as a continuous variable. We assessed relationships between various smoking categories and a risk of abnormal birth weight (birth weight <10th percentile, fetal growth restriction, and birth weight <2500 g) and other adverse birth outcomes. We assessed the whole cohort and the subgroups of various categories of pre-pregnancy BMI, as well as the subgroup of women who did not develop hypertension or diabetes in the current pregnancy. We conducted this analysis in a prospectively collected cohort.

We calculated odds ratios (and statistical power) of the birth outcomes in multidimensional logistic regression. Analyses like ours have not been reported very often. To our knowledge, this is one of the first such studies in Poland.

## 2. Experimental Section

In this analysis, we evaluated a cohort of 912 mother and newborn pairs. It was a prospectively collected cohort of pregnant women, and the study was conducted at the Obstetrics and Gynecology Hospital of the Medical University in Poznań (Poland); it is the center of the highest (third degree) reference for obstetrics and neonatology. The recruitment of women who were at the end of the first trimester of pregnancy was carried out in 2015–2016, and the pregnancy results of all the participants were recorded in 2016–2017.

The objectives and design of the study were developed in accordance with the Helsinki Declaration, and were adopted by the Bioethics Committee operating at the Medical University of Poznań, Poland (769/15 and 1092/15). All the women were informed about the voluntary participation in the study and the aims of the study. All of them expressed and signed their informed consent.

### 2.1. Method

The recruitment of the participants for the presented study was carried out among pregnant women reporting to the hospital laboratory for routine tests. Brief information about the study was posted in the laboratory to be visible to all patients.

The recruitment criteria for the presented cohort included women aged 18–45 (at the conception), healthy single pregnancy (without aneuploidy), gestational age during recruitment of 10–14th week, and delivery in ≥25th gestational week. Women without any chronic diseases were recruited, the exception being abnormal body mass index values. The use of multi-vitamin-micronutrient or folic acid preparations typical for pregnancy was not used as an inclusion or exclusion criterion.

During the recruitment period, a total of 1300 women declared their readiness to participate in the study in question over 12 months. After delivery, 388 women were excluded due to the development of hypertension or diabetes before 20th and 18th week (respectively), delivery of a newborn with a birth defect and/or delivery before 25th week, as well as due to incomplete data. Finally, a cohort of 912 mother and child pairs was qualified for further analysis.

The first stage of the study (during recruitment) consisted in completing a questionnaire containing questions about sociodemographic and economic data, about the course of the current pregnancy and the history of previous pregnancies, about previous diseases and diseases in the family, as well as about lifestyle, including smoking. The women completed the questionnaire themselves, in the presence of the midwives who clarified any possible doubts. All the participants declared no use of drugs, electronic cigarettes, and alcohol during pregnancy.

The next stage of the study was collecting information about the results of pregnancy, which was taken from medical records. The data included child outcomes and maternal complications, as well as information about addictions (including smoking). Each participant was contacted after the end of puerperium (on the phone or by mail) to obtain additional information regarding possible change in the smoking habit in pregnancy and the course of puerperium (“additional questionnaire”).

In the presented cohort (*N* = 912), we recorded the following results: 72 (7.9%) women gave birth to newborns with birth weight <10th percentile, 741 (81.2%) women gave birth to newborns between 10th and 90th percentile, 99 (10.9%) women gave birth to newborns >90th percentile, 60 (6.6%) women gave birth to newborns with birth weight <2500 g, 755 (82.8%) women gave birth to newborns between 2500 and 4000 g, 97 (10.6%) women gave birth to newborns >4000 g, 65 (7.1%) women gave birth to a child before 37th week, and 382 (41.9%) women gave birth by Cesarean section (regardless of the reason). Fetal growth restriction (FGR) was diagnosed in 21 (2.3%) newborns, and preeclampsia was diagnosed in 24 (2.6%) women.

### 2.2. Definitions of Dependent Variables

The major dependent variables used in this study were the categories of lower birth weight and fetal growth restriction. Birth weight was registered in medical documentation. The weight of the newborns immediately after birth was measured using an electronic balance and expressed in grams. The birth weight assessment in percentiles was based on Polish percentile grids appropriate for fetal sex and gestational age [13].

Low birth weight (LBW) included newborns <2500 g (*n* = 60). Birth weight <10th percentile (*n* = 72) included both constitutionally small newborns (though healthy, with appropriate intrauterine growth potential) (*n* = 56) as well as newborns diagnosed with fetal growth restriction (*n* = 16) confirmed after birth.

Fetal growth restriction (FGR) was diagnosed based on ultrasound in pregnancy (all cases were recorded, regardless of phenotype) [14,15,16]. FGR was defined as the fetus’ inability to reach its full growth potential and was diagnosed based on ultrasound in pregnancy. The diagnostics included determination of the pregnancy age in the first trimester based on the crown-rump length, serial biometric measurements in order to establish growth potential for an individual fetus, and doppler examination of the umbilical artery. Medical documentation results were recorded during the study and all pregnancies with diagnosed FGR (*n* = 21) were included in these analyses, including 16 cases with placenta dysfunction being the cause (and with confirmation of post-birth FGR diagnosis and birth weight <10th percentile) as well as five cases with birth weight > 10th percentile (post-birth). Detailed information on criteria used for diagnosis is unknown and likely varied by physician [14].

Measurement of gestational age was based on ultrasound examination in the first trimester; the basis for assessment at 10 (+0)–13 (+6 days)th week was crown-rump length (CRL), and above the 14th week, a combination of different measurements was taken into account.

Major pregnancy complications in the mother were noted from medical records. Hypertension developing in pregnancy (according to [27] and Polish guidelines from 2015) was defined as arterial pressure ≥140/90 mmHg, which developed after the 20th gestational week and receded within 12 weeks after delivery. These pressure values were obtained in two measurements and 4 h apart; measurements were taken in a sitting position, with an oscillometric device used for the measurement. When hypertension was accompanied by organ disorders (affecting the kidneys or/and liver or/and thrombocytopenia or/and the brain or/and lungs) meeting diagnostic criteria, preeclampsia (PE) was diagnosed. In our cohort, arterial hypertension (developed de novo after 20th week) and de novo proteinuria ≥0.3 g/L were found in all PE cases. In our study, FGR was not a criterion for the diagnosis of PE. When hypertension was not associated with other organ disorders, gestational hypertension (GH) was diagnosed.

Diabetes developing in pregnancy (according to [28] and Polish guidelines) was diagnosed after using the oral glucose tolerance test (OGTT), carried out between 24 and 28 weeks. It was a 2-h test after loading 75 g of glucose, following an overnight fast. Diabetes that was only treated by a diet (GDM-1) was diagnosed, as well as insulin-requiring diabetes (GDM-2).

### 2.3. Independent Variables

In this analysis, the risk of adverse newborn outcomes was examined, with 9 smoking categories as independent variables.

Smoking categories were self-reported by pregnant women during the recruitment and after the end of puerperium. Additionally, the information about smoking cigarettes was recorded in the medical documentation. We found that the data included in questionnaires and medical documentation were consistent. There was no woman who started smoking during pregnancy.

The following categories of smoking reported by women in the questionnaires were included: the length of cigarette smoking time, number of cigarettes smoked during the day, smoking (ever) before pregnancy, giving up smoking before pregnancy, smoking in the 1st trimester, giving up smoking in the 2nd/3rd trimester, reduction of smoking during pregnancy in the 2nd/3rd trimester, smoking unchanged throughout pregnancy. Additionally, the value of pack-years was calculated for each woman (the quotient of the number of years of smoking and the part of a packet of cigarettes smoked per day, taking 20 cigarettes in a pack).

Clinical factors of LBW, birth weight <10th percentile, and FGR risk were identified based on the literature [17].The variables that were taken into account in the characteristics of mothers included smoking categories, maternal age (years), pre-pregnancy body mass index (BMI), parity, maternal height, gestational weight gain (GWG), prior fetal hypotrophy, hypertension/preeclampsia in previous pregnancy, fetal sex, premature rupture of membranes (PROM), gestation age/preterm birth, and hypertension/preeclampsia in the current pregnancy [29].

In order to conduct subgroup analyses, body mass index (BMI) categories were designated and established for each woman according to the guidelines provided by the World Health Organization. BMI for pre-pregnancy weight was calculated as the quotient of weight (kg) (self-reported) and height (in meters) squared. Normal weight included index 18.5−24.99 kg/m^2^. Underweight included index <18.5 kg/m^2^, overweight 25−29.99 kg/m^2^, and obesity ≥30 kg/m^2^. The information on the mother’s height and pre-delivery weight was taken from medical records. The difference between pre-delivery weight and pre-pregnancy weight was the gestational weight gain (GWG). GWG categories were defined in line with the Institute of Medicine (IOM) from 2009.

### 2.4. Statistical Analyses

Maternal characteristics were compared for the women who never smoked and those who smoked in the 1st trimester, as well as for the women who gave birth to newborns with birth weight <10th percentile and newborns between 10th and 90th percentile. The characteristics of categorical variables are presented using numbers and percentages of individual categories. The characteristics of continuous variables were presented using median (and quartiles) and/or mean values and standard deviation (SD). The normality of the data distribution was tested by the Shapiro–Wilk test. Due to the lack of compliance of continuous variables with the normal distribution, the comparison of two groups was made using the Mann–Whitney test. The Cochran–Armitage test was used to detect a trend in variables representing the division of a continuous variable into ordered categories. Dichotomous variables, depending on the fulfillment of the Cochran condition regarding the number of individual categories, were compared by the Pearson chi-square test when the Cochran condition was met or the Fisher exact if not.

Several analyses were carried out which compared women with the following pregnancy outcomes: fetal growth restriction (FGR) cases vs. no FGR, newborns <10th percentile vs. newborns between 10th and 90th percentile, LBW newborns (<2500 g) vs. newborns 2500–4000 g, newborns >90th percentile vs. newborns between 10th and 90th percentile, newborns >4000 g vs. newborns 2500–4000 g, birth <37th week vs. birth ≥37th week, Cesarean section vs. other deliveries, as well as women who developed preeclampsia (PE) vs. normotensive women.

Statistical analyses assessing the effects of smoking on the risk of birth outcomes were performed using one- and multi-dimensional logistic regression; crude odds ratios (OR) and adjusted odds ratios (AOR) were calculated. The odds ratios of birth outcomes for smoking categories was given in relation to the reference category “women who have never smoked” or “smokers in the 1st trimester” as well as “never smokers with normal BMI”. To describe the risk size, the odds ratio together with the 95% confidence interval, CI, was quoted. The statistical significance of the results in the logistic regression was checked using Wald’s test.

The one-dimensional logistic regression model was rebuilt and also assessed after dissection of the entire cohort into the pre-pregnancy BMI categories and in the subgroup of the women who did not develop hypertension or diabetes in the current pregnancy (“Healthy” women).

The adjusted odds ratio (AOR) was given in multi-dimensional analyses. Four correcting models were used. Correcting variables were chosen from among risk factors provided by the literature (as above) and on the basis of characteristics of women with newborn <10th percentile and 10–90th percentile (mother’s characteristics that were significantly statistically different between these groups were chosen). Model-a (AOR-a) included the following variables: maternal age, pre-pregnancy BMI, maternal height, and gestational age at birth. Model-b (AOR-b) included model-a and prior hypotrophy. Model-c for FGR (AOR-c) included maternal age, pre-pregnancy BMI, maternal height, prior hypotrophy, and preeclampsia in the current pregnancy. Model-c for birth weight <10th percentile and LBW (AOR-c) included maternal age ≥40 years, child birth number ≥3, prior hypotrophy, maternal height, gestational age, hypertension in the current pregnancy. Model-d (AOR-d) included primiparous women, pre-pregnancy BMI, maternal age, gestational weight gain outside the range of the recommendations regardless of the BMI category (for FGR) plus fetal sex, gestational age, maternal height, preeclampsia, and gestational diabetes mellitus (for birth weight <10th percentile and LBW). Only “model-a” (without pre-pregnancy BMI) was used in BMI category analyses (and in the analyses for ideal reference category: never smokers with normal BMI).

Due to the relatively small sizes of the studied groups/subgroups, the analyses also included statistical power (observed power and expected power) (in the study of categorial variables). The power was determined for two situations describing the effect size (odds ratios). In the first situation, the observed power—that is, the power for odds ratios obtained (OR) from the study—was calculated. In the second situation, the expected power was the power determined with an assumption that OR is 2 for risk increasing factors and for risk decreasing factor it is ^1^/_2_. Power 0.5–0.75 is accepted to be the average, whereas power 0.75–1.0 high.

The PQStat v1.8.0 software (PQStat Software Company, Poznan, Poland) was used for the statistical calculations. A significance level of 0.05 was adopted in all analyses. In addition, the results of the analyses, i.e., the odds ratios and 95% CI, were visualized on the charts.

## 3. Results

Table S1 shows that 168 (18.4%) women smoked before pregnancy; the mean number of cigarettes smoked daily per smoker was 10.8 (range 1–30), and the mean length of cigarette smoking time was 8.5 years (range 1–25). Among 57 women who smoked in the first trimester, 22 (38.6%) women quit smoking during the second/third trimester, and 10 (17.6%) women reduced their smoking during the second/third trimester. In the cohort, 29.7% of women were obese or overweight (BMI ≥ 25 kg/m^2^) before pregnancy. In the cohort, 137 women developed hypertension in pregnancy, 146 women developed diabetes in pregnancy, and 654 (71.7%) women did not develop hypertension or diabetes.

Basic characteristics of the women who smoked in the first trimester are presented in Table 1.

The median age, pre-pregnancy BMI, and gestational weight gain (GWG), as well as the number of primiparous women, did not differ between these groups. In the women who smoked in the first trimester, the median newborn weight was statistically significantly lower, the number of newborns <10th percentile was higher (26.3% vs. 7.0%), the number of newborns <2500 g was higher (17.5% vs. 6.2%), and the number of FGR cases was higher (7.1% vs. 2.2%), compared to the women who had never smoked (Table 1). Table S2 shows the general characteristics of women who gave birth to newborns with birth weight <10th percentile.

**Figure 1:**
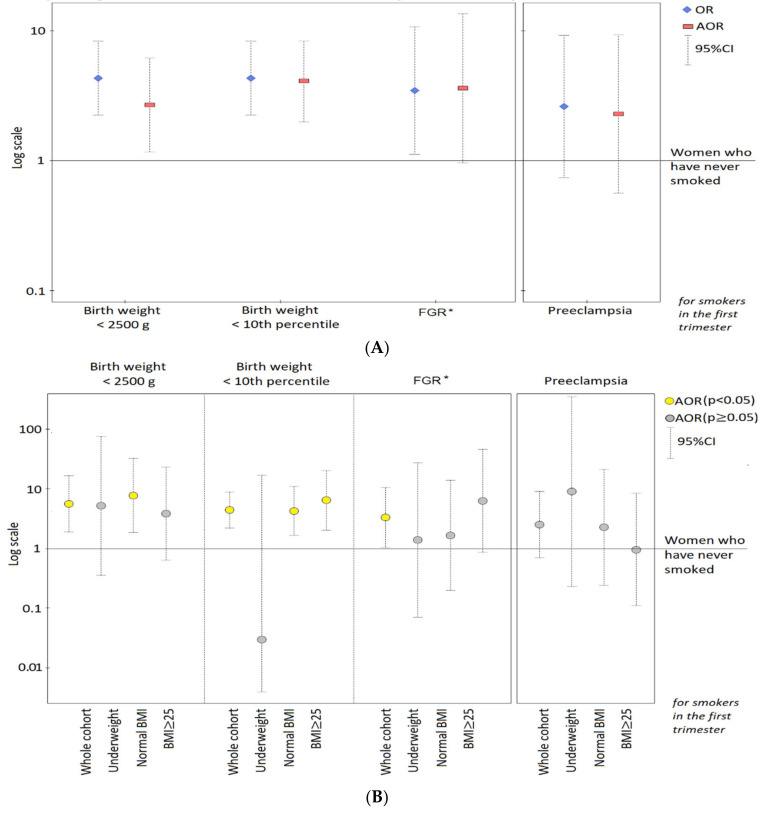
Odds ratios (and 95% confidence intervals) of newborn outcomes (and preeclampsia) for smoking in the 1st trimester. The graphs are presented on a logarithmic scale. * FGR: fetal growth restriction. The adjusted odds ratios (AOR) were calculated after adjusting for: (**A**) primiparous women, maternal age, pre-pregnancy BMI as well as GWG outside the range; (**B**) maternal age, height, pre-pregnancy BMI, and gestational age at birth (excluding gestational age in the FGR study, as well as excluding BMI in the BMI categories). The result for LBW in underweight women is crude.

Figure 1 presents the risk of abnormal birth weight for smoking in the first trimester.

Figure 1 shows that in the whole cohort (A), smoking in the first trimester was associated with higher odds ratios of the outcomes. After dissection of the cohort into pre-pregnancy BMI categories (B), the highest odds ratios of newborns <10th percentile and FGR were found in the excessive BMI subgroup (≥25 kg/m^2^) and the results were statistically significant (yellow points). The results for preeclampsia risk (for comparison with FGR risk) were also presented.

Table 2 and Table S3 show the risk of birth weight <10th percentile, FGR, and LBW newborns for various smoking categories in the one- and multi-dimensional logistic regression. Table S3 shows statistical power (observed/expected) of obtained odds ratios and odds ratios in different correcting models.

Smoking before pregnancy (Table 2 and Table S3), compared to the women who had never smoked, was associated with higher adjusted odds ratios of birth weight <10th percentile (AOR-d = 1.93, CI: 1.08–3.44), FGR (AOR-d = 1.13, CI: 0.38–3.36), and LBW (AOR-d = 2.76, CI: 1.05–7.26). However, only in the birth weight <10th percentile study, the result was statistically significant, and its observed/expected power was average (0.5388/0.7011). At the same time, confidence intervals for the birth weight <10th percentile study were quite narrow. In the FGR study, statistical insignificance results from both the poor effect and the small size of the sample (observed and expected power were low: 0.1209/0.3422 (Table S3)).

Smoking in the first trimester (Table 2 and Table S3), compared to the women who had never smoked, was associated with a 3–6 fold higher adjusted odds ratio of birth weight <10th percentile, FGR, and LBW. Results for birth weight <10th percentile and LBW were statistically significant in all the correcting models and their “observed power” was high (0.9642 and 0.7800, respectively). Confidence intervals were slightly wider than for smokers before pregnancy. Statistical significance of FGR result was sustained after being corrected with maternal age, pre-pregnancy BMI, and maternal height (model-a, Table S3). A value of the observed power higher than the expected one (0.5847/0.2482) shows the role of the small sample size in the FGR analyses.

Smoking cessation before pregnancy (Table 2 and Table S3), compared to the women who had never smoked, was associated with lower adjusted odds ratios of birth weight <10th percentile and FGR: AOR-d = 0.74 (CI: 0.28–1.94) and AOR-d = 0.36 (CI:0.05–2.81) (respectively), but not for LBW (AOR-d = 1.10, CI:0.25–4.83). Indexes of both types of statistical power (observed power and expected power) of the results were low, which implies that statistical insignificance can be caused by a poor effect or a small size of the sample. At the same time, smoking cessation before pregnancy, compared to smoking in the first trimester, was associated with over 5-fold lower odds ratios of birth weight <10th percentile and LBW (statistically significant) and the results were sustained in various correcting models. Observed power for the results of birth weight <10th percentile and LBW was high (0.9737 and 0.8456, respectively) (Table S3).

Smoking cessation in the second/third trimester (Table 2 and Table S3), compared to the women who had never smoked, was associated with 3–5 fold higher adjusted odds ratios of birth weight <10th percentile, FGR, and LBW (to a similar degree to smoking in the first trimester). Only the result for birth weight <10th percentile had a high observed power (0.7742) which was sustained in different correcting models (Table S3). In the FGR study, wide confidence intervals indicate the small size of the sample. All these results can suggest that smoking cessation during pregnancy has no protective effect.

Smoking reduction during the second/third trimester (decrease in cigarettes/daily) intensified the risk of the studied newborn outcomes (Table 2 and Table S3). Smoking reduction in the second/third trimester, compared to the women who had never smoked, was associated with a higher adjusted birth weight <10th percentile risk (AOR-d = 11.91, CI: 2.99–47.5) and LBW risk (AOR-d = 5.19, CI: 1.04–26.05), and observed power was high or average (0.8904 and 0.6735, respectively). These results were statistically significant and sustained after having been corrected in various models, though confidence intervals for these results were wider.

Smoking throughout pregnancy (Table 2 and Table S3), compared to the women who had never smoked, was associated with around 2-fold higher odds ratios for birth weight <10th percentile, FGR, and LBW. These results, however, were statistically insignificant and both demonstrated low power.

The analyses in the BMI categories are presented in Table 3 and Table S4. The coexistence of smoking in the first trimester and maternal obesity or overweight increased the studied risks; the women with excessive weight (BMI ≥ 25 kg/m^2^) had higher adjusted odds ratios of birth weight <10th percentile (AOR-a = 6.39, CI: 2.01–20.34), FGR (AOR-a = 6.25, CI: 0.86–45.59), and LBW (AOR-a = 3.80, CI: 0.63–22.95) for smoking in the first trimester compared to never smokers (Table 3). “Observed power” of the result for birth weight <10th percentile was high (0.8377), whereas the power for FGR was average (0.5318) and the confidence interval was very wide (Table S4).

Table S4 shows the statistical power (observed/expected) of obtained odds ratios in the various subgroups, and the results for “ideal” reference category (never smokers with normal BMI). When the analyses (for smoking in the first trimester) were performed for the “ideal” reference category, we found significantly decreased adjusted odds ratios of birth weight <10th percentile (AOR-a = 1.60, CI: 1.2–2.14), FGR (AOR-a = 1.52, CI: 0.98–2.38), and LBW (AOR-a = 1.58, CI: 1.07–2.33) in the subgroup of BMI ≥25 kg/m^2^ (Table S4).

The coexistence of maternal underweight and smoking in the first trimester increased the FGR risk, compared to never smokers with normal BMI (AOR = 22.42, CI:1.62–310.26, *p* = 0.020).

In the subgroup of the “healthy” women (without hypertension and diabetes), the results for birth weight <10th percentile, FGR, and LBW were similar to the results in the whole cohort (Table S4).

Table 4 shows that longer smoking periods (but not the number of cigarettes smoked per day) increased the adjusted odds ratios of birth weight <10th percentile, FGR, and LBW. In the multidimensional logistic regression (in Model-d), extending smoking time by 1 year increased the risk of birth weight <10th percentile by 9% (AOR-d = 1.09, CI:1.04–1.14, *p* = 0.001) and LBW by 13% (AOR-d = 1.13, CI:1.05–1.21, *p* = 0.001). The results for FGR were not statistically significant.

**Figure 2:**
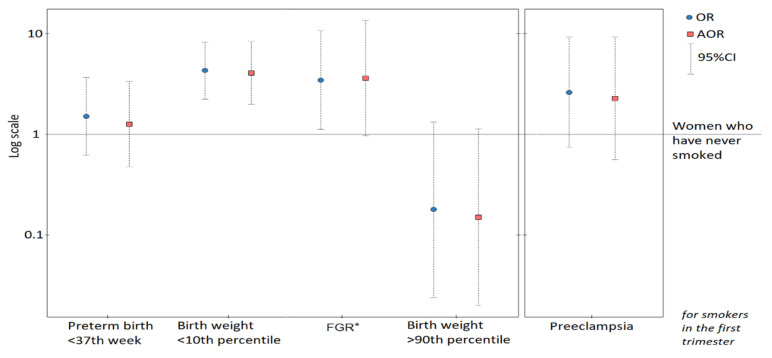
Odds ratios (and 95% confidence intervals) of newborn outcomes (and preeclampsia) for smoking in the 1st trimester. The graphs are presented on a logarithmic scale. The adjusted odds ratios (AOR) were calculated after adjusting for primiparous women, maternal age, pre-pregnancy BMI as well as gestational weight gain outside the range. *FGR: fetal growth restriction.

Figure 2 presents graphic pictures of the odds ratios of other birth outcomes.

Table S5 shows results for other birth outcomes. Smoking in the first trimester was associated with a lower odds ratio of newborns >90th percentile (AOR-d = 0.15, CI:0.02–1.15, *p* = 0.068). The relationships with preterm birth <37th week (AOR-d = 1.26, CI:0.47–3.33, *p* = 0.648) or Cesarean section (AOR-d = 1.09, CI:0.61–1.97, *p* = 0.764) were insignificant.

## 4. Discussion

In this prospective study, we assessed a cohort of 912 mother–child pairs, in order to examine the relations between nine smoking categories reported by mothers and the risk of birth weight <10th percentile, fetal growth restriction (FGR), and LBW (birth weight <2500 g). Many of the results of this detailed analysis were found to have a high statistical power (especially for birth weight <10th percentile risk). The studied groups/subgroups, however, were often small and some confidence intervals were wide; therefore, our conclusions need to be cautious.

Firstly, our results can suggest that smoking in the first trimester involves a risk for lower birth weight. Secondly, it is possible that smoking cessation or reduction in the second/third trimester has no protective effect or can even intensify the analyzed risks. At the same time, our results can imply a protective effect of smoking cessation before pregnancy, particularly in comparison with those who smoked in the first trimester. Thirdly, combined effects of smoking in the first trimester and obesity/overweight are also possible. Analyses in various BMI categories and differences in the odds ratios (for smoking in the first trimester), as compared to the reference category of never smokers or those without obesity, can suggest a strong impact of obesity/overweight on the studied risks, as well as a strong impact of underweight on FGR risk. Fourthly, we have found that a longer time of smoking increased the considered risks. Importantly, high odds ratio values in some analyses and their high statistical power means that our results cannot be accidental.

Our results for birth weight are in line with previously published studies. Many studies, including the meta-analyses by Abraham et al., showed relationships of smoking during pregnancy with lower fetal measurements, including low birth weight (LBW), small-for-gestational age birth weight (SGA), and FGR [5,17,18,30,31]. Xaverius et al. showed that the risk of low birth weight for quitting smoking before pregnancy was similar to that of never smokers [30]. Our effects of smoking on other adverse pregnancy outcomes are also similar to results noted in the literature [17,23,32]. Contrary to our results, other authors have not confirmed our additive effects of maternal obesity; however, the number of the studies is small [21,22,23]. Studies available in the literature differ regarding the size of the sample, the level of risk in the women surveyed, or a different way of collecting data (retrospectively or prospectively).

In our study, the number of smokers was low. This may suggest the beneficial effects of various government campaigns to reduce this addiction [33]. In our entire cohort, smoking before pregnancy concerned 18.4% of women, and smoking in the first trimester 6.3%. Out of the 57 women who smoked in the first trimester, 22 women quit smoking in the second/third trimester, and 10 women reduced smoking during the second/third trimester. Women self-reported smoking but the information has been verified two times. In our entire cohort, the frequency of birth weight <10th percentile and LBW was 7.9% and 6.6% (respectively).

Many our results for FGR were statistically significant, but this case group was the smallest (*n* = 21), and the statistical power (especially expected power) was small for all the results. Confidence intervals in FGR risk were wide, indicating the role of the sample small size. Importantly, in this study, we have examined all the FGR (n = 21) cases diagnosed based on ultrasound (USG) examination during pregnancy, 16 (76%) of which were identified after birth as growth potential restriction of a fetus with birth weight <10th percentile. Our approach could have contributed to the lack of statistical significance of some results for FGR. As compared to the results for FGR, smoking in the first trimester also increased the risk of preeclampsia (PE), but in a weaker and statistically insignificant way (Figure 1, Table S4).

Importantly, our analyses show that smoking cessation in the second/third trimester was associated with a higher risk of studied outcomes (including FGR) to a similar degree to smoking in the first trimester (Table 2). Contrary to the results of this analysis, our previous study in the same cohort showed that quitting smoking in the second/third trimester increased the risk of pregnancy hypertension (GH) and preeclampsia (PE) much more than smoking in the first trimester [20]. In the current analysis, we found a greater risk of presented outcomes in the women who reduced smoking in the second/third trimester, but this subgroup was very small (*n* = 10) and we treat this part of the analysis as a pilot study that requires further investigation (Table S3).

The comparison of our results from this and our previous research is important, because FGR often coexists with PE, and in the pathogenesis of both cases, the role of placental ischemia resulting from abnormal spiral arterial transformation in early pregnancy is taken into consideration [9,10,15]. In our cohort, 18.2% of the women who developed PE and 1.4% of women who remained normotensive (*p* < 0.0001) gave birth to newborns with FGR diagnosed during pregnancy. In our cohort, the number of PE cases was also small (n = 24 (2.6%)), and it is consistent with the recruitment criteria for the cohort; during recruitment, we excluded chronic diseases (e.g., hypertension) and multiple pregnancies that increase the risk of preeclampsia.

We showed that the coexistence of smoking in the first trimester with excessive weight of the mother before pregnancy (BMI index ≥25 kg/m^2^) increased the risk for birth weight <10th percentile and FGR (Table 3). Chattrapiban et al. and Heinz-Partington et al. found that maternal obesity softened the effect of cigarette smoking on lower birth weight, and they explained this by the associations of maternal obesity with fetal macrosomia [21,22]. The intensification of smoking effects obtained in our study in women with obesity/overweight may be consistent with our previous articles; we presented that maternal obesity was associated not only with fetal macrosomia, but also with lower birth weight [25,34]. Importantly, in the current study, an analysis of “an ideal reference category” (never smokers with normal BMI) performed after division into BMI categories showed changes in the odds ratios of birth weight <10th percentile, FGR, and LBW in obese/overweight or underweight women. This result can confirm an influence of maternal excessive weight (and underweight) on the studied risks.

The mechanisms of the relationships found (between smoking in the first trimester and the risk of birth weight <10th percentile, LBW, and FGR) are not fully understood. The main factors affecting fetal growth include the placenta, which guarantees the flow of nutrients to the fetus [14,15,16]. Factors causing hypoxia and reductions of blood flow in the placenta may increase the risk of FGR, SGA, and LBW [2,7]. Mechanisms of an increase or decrease in studied risk have not been univocally explained, like the processes of risk intensification in women who give up smoking in the second/third trimester. Some mechanisms may increase, while others may reduce the risks in smoking women in the first trimester (as described below).

Tobacco smoke contains several thousand chemicals, including many toxic substances that exacerbate inflammation and oxidative stress, which can be harmful to placental development and/or be passed through the placenta to the fetus [3,14]. Different chemicals in tobacco smoke can have different effects, and each of the substances can affect the course of several different processes. An example could be nicotine.

On the one hand, in in vitro tests, nicotine disturbs the transformation of spiral arteries, which increases the risk of pregnancy complications [4,9,10]. On the other hand, nicotine may have a beneficial inhibitory effect on thromboxane production and also has anti-inflammatory and immune suppressive effects [4,9,10,11]. Although nicotine has these beneficial effects, it must be remembered that it is also a neuroteratogen and adversely affects the brain of the fetus [3,4]. Nicotine is only one of many compounds, and nitrosamines and hydrocarbons contained in cigarette smoke are genotoxic and have carcinogenic and teratogenic effects. Carbon monoxide (CO) or nitrous oxide (NO) exhibit vasodilatation and exogenous CO can be immunosuppressive. On the other hand, CO can also block oxygen access [3,9]. Toxic substances from tobacco smoke accumulate in the body and the placenta and may increase oxidative stress and reduce antioxidant levels. In our previous studies, we found relationships between lower levels of selenium (a strong antioxidant) in the maternal serum in the first trimester and a higher risk of FGR [12] and a higher risk of birth weight <10th percentile [13]. Relationships between smoking in the first trimester and lower maternal serum selenium levels at the end of the first trimester [12,27], as well as between maternal obesity/overweight and lower maternal selenium levels, were also found [12,27]. There is evidence of oxidative damage to lipids and proteins in smokers, and an increase in oxidative damage to DNA has been found in various biological materials [35]. DNA methylation that mediates between smoking and lower birth weight can also be a factor linking this unfavorable fetal outcome with adverse long-term health outcomes [35,36]. It should also be taken into account that smoking women may lead a different lifestyle that includes, for example, an unhealthy diet and other “unhealthy” behaviors [37].

The mechanisms of the combined effect of obesity and smoking are not fully explained. Relations between obesity and FGR, low birth weight (LBW), or preeclampsia were found in literature studies; this can suggest that obesity contributes to placental blood flow impairment [24]. The stronger smoking effect (on adverse pregnancy outcomes) in obese women could be also explained by the severity of inflammation and oxidative stress that accompanies both smoking and obesity/overweight [3,12,13,14,27,38,39].

The major clinical implications of this study relate to the adverse effects of smoking in the first trimester and the result which confirms that a longer time of smoking increases the risk of lower birth weight. In the current study, only smoking cessation before pregnancy was associated with lower chances of studied outcomes; results for this smoking category compared to smokers in the first trimester had high statistical power and statistical significance. Confirmation (in other studies) of the paradoxical intensification of the risk of adverse fetal outcomes in women who reduced their smoking during pregnancy implies caution in making such decisions. Smoking, however, has many adverse effects; therefore, even a lack of protective effect in women who gave up smoking in the second/third trimester can turn out to be beneficial.

### Limitations and Benefits

An advantage of the current analysis is the prospective model of the study (in this model, the final results of pregnancy are unknown during recruitment). We have assessed several smoking categories. We adjusted our results for many risk factors; however, it is possible that there are other confounders that affect our results. The participants came from one region, which means that study and control groups were matched in terms of diet (common to the region) and level of prenatal care.

The limitation of our study was the lack of information about passive smoking. The reduction in the frequency of smoking among women meant that in a large enough prospective cohort, there were few women in subgroups, e.g., women who reduced their smoking during pregnancy. However, despite the small quantity of some subgroups, the statistical power of many results was high. In our study, smoking was self-reported by pregnant women but the information obtained during recruitment was verified after the end of pregnancy (and puerperium). Additionally, the information obtained by means of questionnaires was compared with the information included in medical documentation; ultimately, there was no discrepancy between information from the three sources.

## 5. Conclusions

Our results can suggest that smoking in the first trimester involves a risk for birth weight <10th percentile, fetal growth restriction (FGR), and LBW (birth weight <2500 g). It is possible that smoking cessation or reduction in the second/third trimester has no protective effect or can even intensify the analyzed risks. Nevertheless, other aspects of the harmful effects of smoking on fetal development need to be remembered. At the same time, our results suggest that smoking cessation before pregnancy could be the only safe strategy for reduction of studied risks. We have also found that a longer time of smoking increased the considered risks.

All women should avoid smoking during the first trimester, with special attention and support for the high-risk group of women with non-optimal BMI.

Our studied groups/subgroups were often small; however, high odds ratio values in some analyses and their high statistical power mean that our results cannot be accidental. Future prospective studies in larger groups are recommended.

## Figures and Tables

**Table 1:** Basic characteristics of the women who smoked in the 1st trimester.

Maternal Characteristics/ Pregnancy Outcomes	Women Who Have Never Smoked (N = 744)	Smokers in 1st Trimester (N = 57)	*p* *
	Median (IQ Range)/n (%)	Median (IQ Range)/n (%)	
Maternal age (years)	35 (31–37)	34 (27–38)	0.473
Primiparous	311 (41.8%)	19 (33.3%)	0.211
Gestational weight gain (kg)	13 (10–17)	14 (10–20)	0.220
Pre-pregnancy BMI (kg/m^2^)	22.6 (20.4–25.8)	23.6 (21.3–27.7)	0.078
Education <12 years (available data)	42 (6.6%)	17 (31.5%)	<0.001
Fetal sex, son	386 (51.9%)	29 (50.9%)	0.884
Fetal growth restriction (FGR)	16 (2.2%)	4 (7.1%)	0.047
Birth weight (g)	3400 (3080–3722.5)	3350 (2740–3550)	0.023
Birth weight in percentiles			<0.001
<10th	52 (7.0%)	15 (26.3%)	
10–90th	609 (81.9%)	41 (71.9%)	
>90th	83 (11.2%)	1 (1.8%)	
Birth weight in grams			0.003
<2500	46 (6.2%)	10 (17.5%)	
2500–4000	618 (83.1%)	44 (77.2%)	
>4000	80 (10.8%)	3 (5.3%)	
Preeclampsia (PE)	20 (2.7%)	3 (5.3%)	0.221
APGAR in the 5th min <7	2 (0.3%)	0 (0%)	1
Birth <37th week	54 (7.3%)	6 (10.5%)	0.428
Cesarean section	313 (42.1%)	24 (42.1%)	0.996

* The Mann–Whitney U test was used for comparisons of continuous variables, the Cochran–Armitage test was used to detect a trend, and for binomial categories, the Pearson chi-square test (or Fisher exact test when Cochran assumption was not met) was used (*p*-value < 0.05 was assumed to be significant). APGAR is an assessment of appearance, pulse, grimace, activity, and respiration.

**Table 2:** The adjusted odds ratios of birth weight <10th percentile, FGR, and LBW for smoking categories.

Newborn Outcomes/ Smoking Categories	Odds Ratios of Birth Weight for Smoking Categories		
	Cases/ Controls	OR (95% CI:); *p*	AOR-d * (95% CI:); *p*
Birth weight <10th percentile			
Smoking before pregnancy	20/132	1.77 (1.03–3.07); 0.041	1.93 (1.08–3.44); 0.027
Smoking cessation before pregnancy	5/91	0.64 (0.25–1.65); 0.360	0.74 (0.28–1.94); 0.541
Smoking in 1st trimester	15/41	4.29 (2.22–8.26); <0.001	4.68 (2.28–9.62); <0.001
Smoking cessation in 2nd/3rd trimester #	6/16	4.39 (1.65–11.7); 0.003	4.54 (1.58–13.02); 0.005
Reduction of smoking in 2nd/3rd trimester #	5/5	11.71 (3.28–41.77); <0.001	11.91 (2.99–47.5); <0.001
Smoking unchanged #	4/20	2.34 (0.77–7.11); 0.133	2.55 (0.77–8.42); 0.126
Women who have never smoked	52/609	1	1
Smoking cessation before pregnancy	5/91	0.15 (0.05–0.44); 0.001	0.17 (0.05–0.52); 0.002
Smoking in 1st trimester	15/41	1	1 **
FGR			
Smoking before pregnancy	5/162	1.39 (0.50–3.84); 0.531	1.13 (0.38–3.36); 0.822
Smoking cessation before pregnancy	1/110	0.41 (0.05–3.11); 0.387	0.36 (0.05–2.81); 0.327
Smoking in 1st trimester	4/52	3.45 (1.11–10.7); 0.032	3.60 (0.96–13.49); 0.057
Smoking cessation in 2nd/3rd trimester #	2/20	4.49 (0.97–20.84); 0.055	3.36 (0.6–18.74); 0.167
Reduction of smoking in 2nd/3rd trimester #	1/9	4.99 (0.6–41.73); 0.138	6.29 (0.44–90.29); 0.176
Smoking unchanged #	1/23	1.95 (0.25–15.35); 0.525	5.74 (0.57–58.16); 0.139
Women who have never smoked	16/718	1	1
Smoking cessation before pregnancy	1/110	0.12 (0.01–1.08); 0.059	0.12 (0.01–1.15); 0.066
Smoking in 1st trimester	4/52	1	1 **
LBW			
Smoking before pregnancy	14/137	1.37 (0.73–2.57); 0.321	2.76 (1.05–7.26); 0.039
Smoking cessation before pregnancy	4/93	0.58 (0.20–1.64); 0.303	1.10 (0.25–4.83); 0.904
Smoking in 1st trimester	10/44	3.05 (1.44–6.46); 0.003	6.42 (1.84–22.36); 0.004
Smoking cessation in 2nd/3rd trimester #	4/17	3.16 (1.02–9.78); 0.046	2.14 (0.62–7.36); 0.229
Reduction of smoking in 2nd/3rd trimester #	3/7	5.76 (1.44–23.01); 0.013	5.19 (1.04–26.05); 0.045
Smoking unchanged #	3/20	2.02 (0.58–7.03); 0.272	3.18 (0.8–12.64); 0.100
Women who have never smoked	46/618	1	1
Smoking cessation before pregnancy	4/93	0.19 (0.06–0.64); 0.007	0.22 (0.05–1.02); 0.053
Smoking in 1st trimester	10/44	1	1 **

* AOR-d: adjusted odds ratios (and confidence intervals) calculated in the multidimensional logistic regression (*p*-value was calculated in the Wald test and *p* < 0.05 was assumed to be significant); the odds ratios were adjusted for: primiparous women, pre-pregnancy BMI, maternal age, gestational weight gain outside the range of the recommendations regardless of the BMI category (for FGR risk) plus fetal sex, gestational age, maternal height, preeclampsia, gestational diabetes mellitus (for birth weight <10th percentile and LBW risk); ** the odds ratios were obtained after being adjusted (model-a) for maternal age, pre-pregnancy BMI, maternal height (for FGR) plus gestational age at birth (for birth weight <10th percentile and LBW); #: the adjusted odds ratios of newborn outcomes for the variable were adjusted for primiparous women, pre-pregnancy BMI, maternal age and gestational weight gain outside the range (due to the small number of cases). FGR: fetal growth restriction; LBW: birth weight <2500 g.

**Table 3:** The adjusted odds ratios of newborn outcomes for smoking in the 1st trimester, calculated in the subgroups of BMI categories.

	Birth Weight <10th Percentile	FGR	LBW
BMI Categories/ Smoking Categories	OR (95% CI); *p* AOR-a * (95% CI); *p*	OR (95% CI); *p* AOR-a * (95% CI); *p*	OR (95% CI); *p* AOR-a * (95% CI); *p*
Whole cohort (*n* = 801)			
Smoking in 1st trimester	4.29 (2.22–8.26); <0.001	3.45 (1.11–10.7); 0.032	3.05 (1.44–6.46); 0.003
	4.43 (2.21–8.85); <0.001	3.29 (1.02–10.58); 0.046	5.58 (1.88–16.5); 0.002
Never smokers	1	1	1
Underweight (*n* = 39)			
Smoking in 1st trimester	1.50 (0.13–17.04); 0.744	2.58 (0.21–31.2); 0.455	5.17 (0.36–75.13); 0.229
	0.03(0–13.68); 0.259	1.38(0.07–27.17); 0.833	NA *
Never smokers	1	1	1
Normal BMI (*n* = 534)			
Smoking in 1st trimester	3.95 (1.58–9.88); 0.003	1.75 (0.22–14.13); 0.601	3.53 (1.24–9.99); 0.018
	4.25 (1.64–11.0); 0.003	1.66 (0.2–13.81); 0.638	7.67 (1.84–31.9); 0.005
Never smokers	1	1	1
Overweight (*n* = 146)			
Smoking in 1st trimester	6.00 (1.51–23.84); 0.011	NA	5.10 (1.12–23.16); 0.035
	6.95 (1.54–31.27); 0.012	NA	7.84 (0.68–91.08); 0.1
Never smokers	1	1	1
Obesity (*n* = 82)			
Smoking in 1st trimester	4.60 (0.87–24.32); 0.072	6.48 (0.92–45.57); 0.061	0.46 (0.05–4.03); 0.479
	6.42 (0.94–44.08); 0.058	5.12 (0.6–43.63); 0.136	NA *
Never smokers	1	1	1
BMI ≥ 25 kg/m^2^ (*n* = 228)			
Smoking in 1st trimester	5.53 (1.91–15.97); 0.002	6.60 (1.04–41.86); 0.045	1.92 (0.58–6.34); 0.284
	6.39 (2.01–20.34); 0.002	6.25 (0.86–45.59); 0.071	3.80 (0.63–22.95); 0.145
Never smokers	1	1	1

* AOR-a: adjusted odds ratios (with 95% confidence intervals, CI) calculated in the multidimensional logistic regression; the results were adjusted for: maternal age, maternal height, pre-pregnancy BMI and gestational age at birth (excluding gestational age in the study for FGR, as well as excluding BMI in the BMI category analyses); *p*-value was calculated in the Wald test (*p* < 0.05 was assumed as significant). NA: the lack of cases or controls; NA *: impossible to correct (small number of cases); BMI: body mass index (pre-pregnancy values): Underweight: <18.5 kg/m^2^, Normal BMI: 18.5–24.9 kg/m^2^, Overweight: 25.0–29.9 kg/m^2^; Obesity: ≥30 kg/m^2^; FGR: fetal growth restriction; LBW: birth weight <2500 g.

**Table 4:** The adjusted odds ratios of birth weight <10th percentile, FGR, and LBW for smoking categories (continuous variables).

Newborn Outcomes/ Smoking Categories for (Ever) Smokers	OR (95% CI:); *p*	AOR-d * (95% CI:); *p*
Birth weight <10th percentile risk		
Length of smoking time (for 1 year)	1.09 (1.04–1.13); <0.001	1.09 (1.04–1.14); 0.001
Cigarettes/day (for 1 cigarette)	1.01 (0.99–1.03); 0.297	1.01 (0.99–1.03); 0.391
Pack-years (for 1)	1.05 (1.00–1.10); 0.035	1.05 (1.00–1.10); 0.065
FGR risk		
Length of smoking time (for 1 year)	1.07 (1.00–1.16); 0.058	1.08 (0.99–1.17); 0.086
Cigarettes/day (for 1 cigarette)	1.01 (0.97–1.04); 0.817	1.00 (0.95–1.05); 0.989
Pack-years (for 1)	1.02 (0.95–1.10); 0.530	1.02 (0.94–1.10); 0.611
LBW risk		
Length of smoking time (for 1 year)	1.07 (1.02–1.13); 0.006	1.13 (1.05–1.21); 0.001
Cigarettes/day (for 1 cigarette)	1.01 (0.98–1.03); 0.564	1.02 (0.99–1.05); 0.140
Pack-years (for 1)	1.04 (1.00–1.09); 0.077	1.08 (1.02–1.14); 0.012

* AOR-d: adjusted odds ratios (and confidence intervals) calculated in the multidimensional logistic regression (p-value was calculated in the Wald test and *p* < 0.05 was assumed to be significant); the odds ratios were adjusted for: primiparous women, pre-pregnancy BMI, maternal age, gestational weight gain outside the range of the recommendations regardless of the BMI category (for FGR) plus fetal sex, gestational age, maternal height, preeclampsia, gestational diabetes mellitus (for birth weight <10th percentile and LBW). FGR: fetal growth restriction; LBW: birth weight <2500 g.

## Supplementary Materials

The following are available online at https://www.mdpi.com/2077-0383/9/11/3504/s1, Table S1: Smoking categories among smokers, Table S2: Basic characteristics of the women who gave birth to newborn with birth weight <10th percentile, Table S3: The odds ratios (and statistical power) of birth weight <10th percentile, FGR, and LBW for smoking categories in different correcting models, Table S4: The odds ratios (and statistical power) of newborn outcomes for smoking in the 1st trimester, calculated in subgroups, Table S5: The adjusted odds ratios of all newborn outcomes and preeclampsia for main smoking categories.
