# Supplementary material for: Smoking and Smoking Cessation in the Risk for Fetal Growth Restriction and Low Birth Weight and Additive Effect of Maternal Obesity
Source: J Clin Med. 2020 Oct 29;9(11):3504. doi: 10.3390/jcm9113504 (PMC7692695; doi:10.3390/jcm9113504)
Supplement: Supplementary file 1 [file jcm-09-03504-s001.zip › Table S1.docx]

**Table S1.** Smoking categories among smokers.

|  | **Women who**  **have never smoked**  **(N = 744)** | **Smokers**  **(N = 168)** |  | |
| --- | --- | --- | --- | --- |
|  | **Mean (min-max )/ n(%)** | **Mean (min-max) / n(%)** | | ***p*** |
| Women who smoked before pregnancy | - | 168 | | - |
| Length of smoking time (years) | - | 8.5 (1-25) | | - |
| Number of cigarettes/ day (n) | - | 10.8 (1-30) | | - |
| Women who quit smoking before pregnancy | - | 111 | | - |
| Smokers in 1^st^ trimester | - | 57 | | - |
| Women who quit smoking in 2^nd^/3^rd^ trimester | - | 22 | | - |
| Reduction of smoking in 2^nd^/3^rd^ trimester | - | 10 | | - |
| Smoking unchanged until the end of pregnancy | - | 25 | | - |
