# Supplementary material for: Smoking and Smoking Cessation in the Risk for Fetal Growth Restriction and Low Birth Weight and Additive Effect of Maternal Obesity
Source: J Clin Med. 2020 Oct 29;9(11):3504. doi: 10.3390/jcm9113504 (PMC7692695; doi:10.3390/jcm9113504)
Supplement: Supplementary file 1 [file jcm-09-03504-s001.zip › Table S2.docx]

**Table S2.** Basic characteristics of the women who gave birth to newborn with birth weight < 10th percentile.

| **Maternal characteristics /**  **Pregnancy outcomes** | **Women who gave birth**  **to newborns**  **10-90th percentile**  **(n = 741)** | **Women who gave birth**  **to newborns**  **< 10th percentile**  **(n = 72)** |  |
| --- | --- | --- | --- |
|  | **Mean (SD) or n (%)** | **Mean (SD) or n (%)** | **p*** |
| Maternal age (years) | 33.6 (4.7) | 34.0 (5.4) | 0.239 |
| Age ≥40 years (vs others) | 52 (7.0%) | 11 (15.3%) | 0.012 |
| Primiparous women | 316 (42.7%) | 34 (47.2%) | 0.454 |
| Parity ≥3 (vs others) | 28 (3.8%) | 8 (11.1%) | 0.010 |
| Prior fetal hypotrophy | 6 (0.8%) | 6 (8.3%) | <0.0001 |
| Prior GH/PE | 11 (1.5%) | 3 (4.2%) | 0.095 |
| Multivitamins in pregnancy | 427 (57.6%) | 39 (54.2%) | 0.571 |
| Education <12 years (for available data) | 52 (8.0%) | 10 (16.7%) | 0.024 |
| Maternal height (cm) | 166.5 (6.1) | 164.0 (6.6) | 0.004 |
| Pre-pregnancy BMI (kg/m²) | 23.5 (4.2) | 24.1 (5.2) | 0.569 |
| Obesity (BMI ≥ 30) | 64 (8.4%) | 10 (13.9%) | ref |
| Overweight (25.0-29.9) | 137 (18.5%) | 13 (18.1%) |  |
| Normal BMI (18.5-24.9) | 503 (67.9%) | 42 (58.3%) |  |
| Underweight (<18.5) | 36 (4.9%) | 7 (9.7%) | 0.093 |
| Underweight (vs others) | 36 (4.9%) | 7 (9.7%) | 0.078 |
| GWG (kg) | 13.5 (5.7) | 12.5 (6.0) | 0.160 |
| GWG below the range (vs others) | 199 (26.9%) | 26 (36.1%) | 0.094 |
| Smokers ever before pregnancy | 132 (17.8%) | 20 (27.8%) | 0.038 |
| Smokers in the first trimester | 41 (5.5%) | 15 (20.8%) | <0.0001 |
| Fetal sex/ son | 361 (48.7%) | 33 (45.8%) | 0.640 |
| Gestational age at delivery (weeks) | 38.7 (1.9) | 37.9 (2.7) | 0.001 |
| (range) | (25 - 42) | (26 - 42) |  |
| Birth < 37th week | 51 (6.9%) | 11 (15.3%) | 0.010 |
| PROM | 64 (8.6%) | 4 (5.6%) | 0.367 |
| Cesarean section | 300 (40.5%) | 36 (50.0%) | 0.118 |
| Birth weight (g) | 3341.4 (456.3) | 2380.4 (523.9) | <0.0001 |
| (range) | (850.0 – 4300.0) | (495.0 – 3030.0) |  |
| FGR | 5 (0.7%) | 16 (22.5%) | <0.0001 |
| APGAR-5’ <7 | 1 | 0 | - |
| Pregnancy induced hypertension (PIH) | 89 (12.0%) | 30 (41.7%) | <0.0001 |
| Preeclampsia | 14 (1.9%) | 9 (12.5%) | <0.0001 |
| Gestational diabetes mellitus | 108 (14.6%) | 13 (18.1%) | 0.428 |

* The Mann-Whitney U test was used for comparisons of continuous variables, the Cochran-Armitage test was used to detect a trend, and for binomial categories the Pearson chi-square test (or Fisher exact test when Cochran assumption was not met) was used (p-value < 0.05 was assumed to be significant). PROM: premature rupture of membranes; FGR: fetal growth restriction (was diagnosed based on ultrasound in pregnancy); APGAR is an assessment of appearance, pulse, grimace, activity, and respiration.
