# Supplementary material for: Smoking and Smoking Cessation in the Risk for Fetal Growth Restriction and Low Birth Weight and Additive Effect of Maternal Obesity
Source: J Clin Med. 2020 Oct 29;9(11):3504. doi: 10.3390/jcm9113504 (PMC7692695; doi:10.3390/jcm9113504)
Supplement: Supplementary file 1 [file jcm-09-03504-s001.zip › Table S3.docx]

**Table S3.** The odds ratios (and statistical power) of birth weight < 10th percentile, FGR and LBW for smoking categories in different correcting models.

| **Smoking categories** | **OR (95% CI:); *p* Statistical power *** | **AOR-a (95%CI);**  ***p*** | **AOR-c (95%CI);**  ***p*** |
| --- | --- | --- | --- |
| Birth weight < 10th percentile risk |  |  |  |
| Smoking before pregnancy | 1.77 (1.03−3.07); 0.041  0.5388/0.7011 | 1.84 (1.04−3.26); 0.035 | 1.48 (0.81−2.7); 0.200 |
| Smoking cessation before pregnancy | 0.64 (0.25−1.65); 0.360  0.1133/ 0.2066 | 0.70 (0.27−1.83); 0.469 | 0.67 (0.25−1.79); 0.425 |
| Smoking in 1^st^ trimester | 4.29 (2.22−8.26); <0.001  0.9642/ 0.4253 | **4.43 (2.21−8.85); <0.001** | 2.66 (1.26−5.6); 0.010 |
| Smoking cessation in 2^nd^/3^rd^ trimester | 4.39 (1.65−11.7); 0.003  0.7742/ 0.2592 | **4.38 (1.56−12.3); 0.005** | 2.29 (0.78−6.74); 0.133 |
| Smoking reduction in 2^nd^/3^rd^ trimester | 11.71 (3.28−41.8); <0.001  0.8904/ 0.1796 | **11.73 (3.07−44.8); <0.001** | 6.94 (1.48−32.5); 0.014 |
| Smoking unchanged | 2.34 (0.77−7.11); 0.133  0.3835/ 0.2788 | 2.40 (0.76−7.54); 0.135 | 1.98 (0.58−6.69); 0.274 |
| Women who have never smoked | 1 | 1 | 1 |
| Smoking cessation before pregnancy | 0.15 (0.05−0.44); 0.001  0.9737/ 0.3027 | **0.17 (0.05−0.52); 0.002** | 0.23 (0.07−0.75); 0.015 |
| Smoking in 1^st^ trimester | 1 | 1 | 1 |
| IUGR risk |  |  |  |
| Smoking before pregnancy | 1.39 (0.50−3.84); 0.531  0.1209/ 0.3422 | 1.34 (0.48−3.76); 0.577 | 1.13 (0.38−3.4); 0.829 |
| Smoking cessation before pregnancy | 0.41 (0.05−3.11); 0.387  0.0604/ 0.0533 | 0.41 (0.05−3.17); 0.396 | 0.45 (0.06−3.47); 0.443 |
| Smoking in 1^st^ trimester | 3.45 (1.11−10.7); 0.032  0.5847/ 0.2482 | 3.29 (1.02−10.58); 0.046 | 2.19 (0.59−8.06); 0.241 |
| Smoking cessation in 2^nd^/3^rd^ trimester | 4.49 (0.97−20.84); 0.055  0.5434/ 0.1945 | **4.99 (1.01−24.74); 0.049** | 4.87 (0.93−25.38); 0.06 |
| Smoking reduction in 2^nd^/3^rd^ trimester | 4.99 (0.6−41.73); 0.138  0.4757/ 0.1747 | 4.11 (0.45−37.25); 0.209 | 1.39 (0.08−24.52); 0.823 |
| Smoking unchanged | 1.95 (0.25−15.35); 0.525  0.1863/ 0.1943 | 1.82 (0.23−14.71); 0.575 | 1.31 (0.15−11.67); 0.81 |
| Women who have never smoked | 1 | 1 | 1 |
| Smoking cessation before pregnancy | 0.12 (0.01−1.08); 0.059  0.0735/ 0.1415 | 0.12 (0.01−1.15); 0.066 | 0.18 (0.01−2.36); 0.190 |
| Smoking in 1^st^ trimester | 1 | 1 | 1 |
| LBW risk |  |  |  |
| Smoking before pregnancy | 1.37 (0.73−2.57); 0.321  0.1895/ 0.6432 | 2.64 (1.08−6.43); 0.033 | 2.21 (0.87−5.6); 0.095 |
| Smoking cessation before pregnancy | 0.58 (0.20−1.64); 0.303  0.1310/ 0.1786 | 1.08 (0.26−4.56); 0.919 | 1.17 (0.27−5.06); 0.835 |
| Smoking in 1^st^ trimester | 3.05 (1.44−6.46); 0.003  0.7800/ 0.3987 | **5.58 (1.88−16.5); 0.002** | 3.91 (1.21−12.62); 0.023 |
| Smoking cessation in 2^nd^/3^rd^ trimester | 3.16 (1.02−9.78); 0.046  0.5369/ 0.2499 | 3.18 (0.55−18.3); 0.195 | 1.93 (0.28−13.18); 0.501 |
| Smoking reduction in 2^nd^/3^rd^ trimester | 5.76 (1.44−23.01); 0.013  0.6735/ 0.1906 | 10.93 (1.61−74.0); 0.014 | 11.66 (1.27−106); 0.030 |
| Smoking unchanged | 2.02 (0.58−7.03); 0.272  0.2680/ 0.2637 | **5.88 (1.14−30.4); 0.034** | 5.13 (0.98−27.0); 0.054 |
| Women who have never smoked | 1 |  |  |
| Smoking cessation before pregnancy | 0.19 (0.06−0.64); 0.007  0.8456/ 0.2492 | 0.22 (0.05−1.02); 0.053 | 0.38 (0.07−1.98); 0.252 |
| Smoking in 1^st^ trimester | 1 | 1 | 1 |

* Statistical power (observed power/expected power).

OR and AOR: crude and adjusted odds ratios (and confidence intervals) calculated in the multidimensional logistic regression (p-value was calculated in the Wald test and p <0.05 was assumed to be significant).

AOR-a: the odds ratios were adjusted for maternal age, pre-pregnancy BMI, maternal height (for FGR) plus gestational age at birth (for birth weight < 10th percentile and LBW);

AOR-c for FGR: the odds ratios were adjusted for maternal age, pre-pregnancy BMI, maternal height, prior hypotrophy and preeclampsia in the current pregnancy;

AOR-c for birth weight < 10th percentile and LBW: the odds ratios were adjusted for maternal age ≥40 years, child birth number ≥ 3, prior hypotrophy, maternal height, gestational age, hypertension in the current pregnancy;

The bold stem shows the results that persisted after correcting in model-b (model-a plus prior hypotrophy).

FGR: fetal growth restriction (was diagnosed based on ultrasound in pregnancy); LBW: birth weight < 2500 g.
