# Supplementary material for: Smoking and Smoking Cessation in the Risk for Fetal Growth Restriction and Low Birth Weight and Additive Effect of Maternal Obesity
Source: J Clin Med. 2020 Oct 29;9(11):3504. doi: 10.3390/jcm9113504 (PMC7692695; doi:10.3390/jcm9113504)
Supplement: Supplementary file 1 [file jcm-09-03504-s001.zip › Table S4.docx]

**Table S4.** The odds ratios (and statistical power) of newborn outcomes for smoking in the 1^st^ trimester, calculated in subgroups.

|  | | **Odds ratios of birth weight for smoking categories** | | |  |  |
| --- | --- | --- | --- | --- | --- | --- |
|  | **Birth weight < 10th percentile** | | **FGR** | **LBW** | | |
| **Groups and subgroups/**  **Smoking categories** | **Statistical power ***  **OR (95% CI); *p***  **AOR-a** (95% CI); *p*** | | **Statistical power ***  **OR (95% CI); *p***  **AOR-a** (95% CI); *p***** | **Statistical power ***  **OR * (95% CI); *p***  **AOR-a** (95% CI); *p*** | | |
| **Whole cohort**  **(*n* = 801)** |  | |  |  | | |
|  | 0.9642/0.4253 | | 0.5847/0.2482 | 0.5049/0.4702 | | |
| Smoking in 1^st^ trimester | 4.29 (2.22−8.26); <0.001 | | 3.45 (1.11−10.7); 0.032 | 3.05 (1.44−6.46); 0.003 | | |
|  | 4.43 (2.21−8.85); <0.001 | | 3.29 (1.02−10.58); 0.046 | 5.58 (1.88−16.5); 0.002 | | |
| Never smokers | 1 | | 1 | 1 | | |
| Smoking in 1^st^ trimester | 4.75 (2.38−9.45); <0.001 | | 3.76 (1.14−12.42); 0.03 | 3.85 (1.74−8.5); 0.001 | | |
|  | 4.79 (2.33−9.85); <0.001 | | 3.49 (1.03−11.8); 0.044 | 5.90 (2.1−16.6); 0.001 | | |
| Ideal category *** | 1 | | 1 | 1 | | |
| **“Healthy” women**  **(*n* = 543) ** **** |  | |  |  | | |
|  | 0.5895/0.2704 | | 0.5293/0.1879 | 0.2993/0.2556 | | |
| Smoking in 1^st^ trimester | 3.19 (1.14−8.93); 0.027 | | 4.55 (0.93−22.41); 0.062 | 2.16 (0.61−7.63); 0.231 | | |
|  | 3.38 (1.17−9.77); 0.024 | | 6.12 (1.16−32.19); 0.033 | 5.14 (1.16−22.72); 0.031 | | |
| Never smokers | 1 | | 1 | 1 | | |
| Smoking in 1^st^ trimester | 2.49 (0.90−6.92); 0.079 | | 3.37(0.71−16.11); 0.128 | 1.88 (0.54−6.61); 0.325 | | |
|  | 2.80 (0.96−8.15); 0.058 | | 3.84 (0.76−19.42); 0.103 | 4.58 (1.1−19.17); 0.037 | | |
| Ideal category *** | 1 | | 1 | 1 | | |
| **Underweight**  **(*n* = 39)** |  | |  |  | | |
|  | 0.0880/0.1348 | | 0.1876/0.1350 | 0.3125/0.1338 | | |
| Smoking in 1^st^ trimester | 1.50 (0.13−17.04); 0.744 | | 2.58 (0.21−31.2); 0.455 | 5.17 (0.36−75.13); 0.229 | | |
|  | 0.03(0−13.68); 0.259 | | 1.38(0.07−27.17); 0.833 | NA * | | |
| Never smokers | 1 | | 1 | 1 | | |
| Smoking in 1^st^ trimester | 4.32 (0.44−42.72); 0.210 | | 12.23(1.25−119.4); 0.031 | 5.64 (0.57−56.1); 0.140 | | |
|  | 3.69 (0.28−48.19); 0.319 | | 22.42 (1.62−310.26); 0.02 | 0.47 (0−271.64); 0.816 | | |
| Ideal category | 1 | | 1 | 1 | | |
| **Normal BMI**  **(*n* = 534)** |  | |  |  | | |
|  | 0.7778/0.2893 | | 0.1513/0.1926 | 0.6344/0.2597 | | |
| Smoking in 1^st^ trimester | 3.95 (1.58−9.88); 0.003 | | 1.75 (0.22−14.13); 0.601 | 3.53 (1.24−9.99); 0.018 | | |
|  | 4.25 (1.64−11.0); 0.003 | | 1.66 (0.2−13.81); 0.638 | 7.67 (1.84−31.9); 0.005 | | |
| Never smokers | 1 | | 1 | 1 | | |
| **Overweight**  **(*n* = 146)** |  | |  |  | | |
|  | 0.7128/0.1862 | |  | 0.5672/0.1751 | | |
| Smoking in 1^st^ trimester | 6.00 (1.51−23.84); 0.011 | | NA | 5.10 (1.12−23.16); 0.035 | | |
|  | 6.95 (1.54−31.27); 0.012 | | NA | 7.84 (0.68−91.08); 0.1 | | |
| Never smokers | 1 | |  | 1 | | |
| Smoking in 1^st^ trimester | 5.76 (1.69−19.72); 0.005 | | ** | 5.64 (1.44−22.09); 0.013 | | |
|  | 1.76 (1.14−2.7); 0.01 | | NA | 2.20 (1.25−3.89); 0.007 | | |
| Ideal category *** | 1 | | 1 | 1 | | |
| **Obesity**  **(*n* = 82)** |  | |  |  | | |
|  | 0.4616/0.1556 | | 0.5401/ 0.1531 | 0.0699/0.0645 | | |
| Smoking in 1^st^ trimester | 4.60 (0.87−24.32); 0.072 | | 6.48 (0.92−45.57); 0.061 | 0.46 (0.05−4.03); 0.479 | | |
|  | 6.42 (0.94−44.08); 0.058 | | 5.12 (0.6−43.63); 0.136 | NA * | | |
| Never smokers | 1 | | 1 | 1 | | |
| Smoking in 1^st^ trimester | 6.49 (1.55−27.11); 0.010 | | 13.97 (2.57−75.8); 0.002 | 2.12 (0.26−17.56); 0.488 | | |
|  | 1.50 (1.04−2.17); 0.03 | | 1.77 (1.14−2.76); 0.011 | 1.29 (0.72−2.29); 0.394 | | |
| Ideal category *** | 1 | | 1 | 1 | | |
| **BMI ≥ 25 kg/m²**  **(*n* = 228)** |  | |  |  | | |
|  | 0.8377/0.2340 | | 0.5318/0.1573 | 0.2330/0.2548 | | |
| Smoking in 1^st^ trimester | 5.53 (1.91−15.97); 0.002 | | 6.60 (1.04−41.86); 0.045 | 1.92 (0.58−6.34); 0.284 | | |
|  | 6.39 (2.01−20.34); 0.002 | | 6.25 (0.86−45.59); 0.071 | 3.80 (0.63−22.95); 0.145 | | |
| Never smokers | 1 | | 1 | 1 | | |
| Smoking in 1^st^ trimester | 6.05 (2.31−15.9); <0.001 | | 1.01 (23.8−1.97); 0.049 | 3.98 (1.25−12.7); 0.019 | | |
|  | 1.60 (1.2−2.14); 0.002 | | 1.52 (0.98−2.38); 0.065 | 1.58 (1.07−2.33); 0.021 | | |
| Ideal category *** | 1 | | 1 | 1 | | |

* Statistical power: observed power/expected power; ** AOR-a: adjusted odds ratios (with 95% confidence intervals, CI) calculated in the multidimensional logistic regression after adjusted for maternal age, maternal height, pre-pregnancy BMI and gestational age at birth (excluding gestational age in the study for IUGR, as well as excluding BMI in the BMI category analyses and in analyses for “ideal reference category); p−value was calculated in the Wald test (p <0.05 was assumed as significant);

*** Ideal category: never smokers with normal BMI.

“Healthy” women: the women who did not develop hypertension or diabetes in the current pregnancy;

FGR: fetal growth restriction (was diagnosed based on ultrasound in pregnancy); LBW: birth weight < 2500 g.

BMI: body mass index (pre−pregnancy values); Underweight: < 18.5 kg/m², Normal BMI: 18.5-24.9 kg/m², Overweight: 25.0-29.9 kg/m²; Obesity: ≥ 30 kg/m².

NA: the lack of cases or controls; NA*: impossible to correct (small number of cases).
