# Supplementary material for: Smoking and Smoking Cessation in the Risk for Fetal Growth Restriction and Low Birth Weight and Additive Effect of Maternal Obesity
Source: J Clin Med. 2020 Oct 29;9(11):3504. doi: 10.3390/jcm9113504 (PMC7692695; doi:10.3390/jcm9113504)
Supplement: Supplementary file 1 [file jcm-09-03504-s001.zip › Table S5.docx]

**Table S5.** The adjusted odds ratios of all newborn outcomes and preeclampsia for main smoking categories.

|  | **Odds ratios of newborn outcomes for smoking categories** | | | |  |  |
| --- | --- | --- | --- | --- | --- | --- |
|  | | **Cases/**  **controls** | **OR (95% CI:); *p*** | **AOR * (95% CI:); *p*** | | |
| Birth weight < 10^th^ percentile ** | |  |  |  | | |
| Smoking before pregnancy | | 20/132 | 1.77 (1.03−3.07); 0.041 | 1.93 (1.08−3.44); 0.027 | | |
| Smoking cessation before pregnancy | | 5/91 | 0.64 (0.25−1.65); 0.360 | 0.74 (0.28−1.94); 0.541 | | |
| Smoking in 1^st^ trimester | | 15/41 | 4.29 (2.22−8.26); <0.001 | 4.68 (2.28−9.62); <0.001 | | |
| Women who have never smoked | | 52/609 | 1 | 1 | | |
| Birth weight <2500 g ** | |  |  |  | | |
| Smoking before pregnancy | | 14/137 | 1.37 (0.73−2.57); 0.321 | 2.76 (1.05−7.26); 0.039 | | |
| Smoking cessation before pregnancy | | 4/93 | 0.58 (0.20−1.64); 0.303 | 1.10 (0.25−4.83); 0.904 | | |
| Smoking in 1^st^ trimester | | 10/44 | 3.05 (1.44−6.46); 0.003 | 6.42 (1.84−22.36); 0.004 | | |
| Women who have never smoked | | 46/618 | 1 | 1 | | |
| FGR *** | |  |  |  | | |
| Smoking before pregnancy | | 5/162 | 1.39 (0.50−3.84); 0.531 | 1.13 (0.38−3.36); 0.822 | | |
| Smoking cessation before pregnancy | | 1/110 | 0.41 (0.05−3.11); 0.387 | 0.36 (0.05−2.81); 0.327 | | |
| Smoking in 1^st^ trimester | | 4/52 | 3.45 (1.11−10.7); 0.032 | 3.60 (0.96−13.49); 0.057 | | |
| Women who have never smoked | | 16/718 | 1 | 1 | | |
| Preeclampsia (PE) ** ** | |  |  |  | | |
| Smoking before pregnancy | | 4/133 | 0.97 (0.33−2.87); 0.950 | 0.91 (0.29−2.88); 0.872 | | |
| Smoking cessation before pregnancy | | 1/96 | 0.33 (0.04−2.52); 0.288 | 0.31 (0.04−2.40); 0.260 | | |
| Smoking in 1^st^ trimester | | 3/37 | 2.60 (0.74−9.16); 0.136 | 2.51 (0.60−10.54); 0.208 | | |
| Women who have never smoked | | 20/642 | 1 | 1 | | |
| Birth weight >90^th^ percentile ** | |  |  |  | | |
| Smoking before pregnancy | | 16/132 | 0.89 (0.5−1.57); 0.685 | 0.81 (0.44−1.47); 0.482 | | |
| Smoking cessation before pregnancy | | 15/91 | 1.21 (0.67−2.19); 0.529 | 1.11 (0.59−2.09); 0.741 | | |
| Smoking in 1^st^ trimester | | 1/41 | 0.18 (0.02−1.32); 0.091 | 0.15 (0.02−1.15); 0.068 | | |
| Women who have never smoked | | 83/609 | 1 | 1 | | |
| Birth weight >4000 g ** | |  |  |  | | |
| Smoking before pregnancy | | 17/137 | 0.96 (0.55−1.67); 0.881 | 0.82 (0.45−1.49); 0.518 | | |
| Smoking cessation before pregnancy | | 14/93 | 1.16 (0.63−2.14); 0.627 | 1.01 (0.52−1.96); 0.981 | | |
| Smoking in 1^st^ trimester | | 3/44 | 0.53 (0.16−1.74); 0.292 | 0.40 (0.11−1.41); 0.153 | | |
| Women who have never smoked | | 80/618 | 1 | 1 | | |
| Birth <37^th^ week *** | |  |  |  | | |
| Smoking before pregnancy | | 11/157 | 0.90 (0.46−1.75); 0.747 | 0.79 (0.39−1.6); 0.512 | | |
| Smoking cessation before pregnancy | | 5/106 | 0.60 (0.24−1.54); 0.290 | 0.54 (0.21−1.43); 0.218 | | |
| Smoking in 1^st^ trimester | | 6/51 | 1.50 (0.62−3.66); 0.369 | 1.26 (0.47−3.33); 0.648 | | |
| Women who have never smoked | | 54/690 | 1 | 1 | | |
| Cesarean section *** | |  |  |  | | |
| Smoking before pregnancy | | 69/99 | 0.96 (0.68−1.35); 0.813 | 0.96 (0.67−1.37); 0.813 | | |
| Smoking cessation before pregnancy | | 45/66 | 0.94 (0.63−1.41); 0.761 | 0.90 (0.59−1.37); 0.625 | | |
| Smoking in 1^st^ trimester | | 24/33 | 1.00 (0.58−1.73); 0.996 | 1.09 (0.61−1.97); 0.764 | | |
| Women who have never smoked | | 313/431 | 1 | 1 | | |

* AOR: adjusted odds ratios (and confidence intervals) calculated in the multidimensional logistic regression and p-value calculated in the Wald test (p <0.05 was assumed to be significant); ** the odds ratios were adjusted for primiparous women, pre-pregnancy BMI, maternal age, gestational weight gain outside the range of the recommendations regardless of the BMI category, fetal sex, gestational age, maternal height, preeclampsia and gestational diabetes mellitus; ***the odds ratios were adjusted for primiparous women, pre-pregnancy BMI, maternal age, gestational weight gain outside the range of the recommendations regardless of the BMI category; ** ** the odds ratios were adjusted for primiparous women, pre-pregnancy BMI, maternal age, gestational weight gain outside the range of the recommendations regardless of the BMI category, hypertension in previous pregnancy and infertility treatment.

FGR: fetal growth restriction (was diagnosed based on ultrasound in pregnancy).
